# Single-Breath Counting Test to Start Non-Invasive Respiratory Support in COVID-19 Patients: Early Detection and the Eternal Dilemma. Comment on Longhitano et al. Single-Breath Counting Test Predicts Non-Invasive Respiratory Support Requirements in Patients with COVID-19 Pneumonia. *J. Clin. Med.* 2022, *11*, 179

**DOI:** 10.3390/jcm11133588

**Published:** 2022-06-22

**Authors:** Stefano Oldani, Serena Bensai, Antonio M. Esquinas

**Affiliations:** 1 Pulmonology Operative Unit, Department of Specialistic Medicine, GB Morgagni Pierantoni Hospital, 47121 Forlì, Italy; bensai.serena@gmail.com; 2 Intensive Care Unit, Hospital Morales Meseguer, 30008 Murcia, Spain; antmesquinas@gmail.com

We have read this study, in which the predictive role of a single-breath counting test (SBCT) to foresee the need of non-invasive respiratory strategies (NIRS) in patients with COVID-19 has been explored, with great interest. We believe this study provides a great contribution to the early management of pneumonia–COVID-19, especially in the Emergency Department (ED) [1]. In fact, SBCT is easy and swift to perform, and—according to the results—it might also help to detect patients with impending worsening respiratory failure. However, we believe that some methodological questions should be pointed out for an adequate extrapolation of the results.

First, can SBCT be influenced by other conditions? SBCT has been used as a surrogate measure of respiratory muscle strength and has been tested in patients with neuromuscular dysfunctions as an equivalent sign of dyspnea [2,3]. Nevertheless, we believe the use of this tool in the ED might be misleading, since speech speed and respiratory drive might be altered by numerous factors. Firstly, the respiratory drive may be influenced by other conditions such as fever, older age, comorbidities, and even anxiety [4]. Furthermore, chronic respiratory diseases were reported in up to 15% of enrolled patients. However, respiratory diseases display completely different respiratory patterns according to the underlying pathophysiology. Patients affected by obstructive pulmonary disease (e.g., COPD) usually show prolonged expiratory time, whereas patients with restrictive lung diseases (e.g., pulmonary fibrosis) often present a reduced vital capacity. We therefore wonder how different baseline respiratory behaviors may affect the reliability of the SBCT. Thus, it would be advisable to know whether the authors evaluated the neuropsychological status and the burden of comorbidities in the enrolled patients to minimize any confounding factors.

Second question: SBTC or PaCO_2_? According to the results, SBTC appears to be as accurate as low PaCO_2_ in predicting the need for NIRS. It is well established that PaCO_2_ correlates with alveolar ventilation, a parameter linked to the respiratory drive activity and respiratory system health status [5]. Thus, PaCO_2_—just like SBCT—might be influenced by all the aforementioned confounding factors. Nevertheless, PaCO_2_ is an objective parameter, and it is not altered by the patient’s ability to perform the test. We therefore inquire whether this feature could be used instead of SBTC in the early detection of worsening COVID-19 patients. 

Third question: SBTC and other severity scores? Some inflammatory markers and some radiological features have been associated with a higher risk of developing severe COVID-19 [6]. Radiological scores, such as the Brixia Score or the Visual Severity Score, have been developed to assess the burden of disease, and are widely used in the decision-making process. We believe it would have been informative to know if the SBCT results were correlated to other available predictive parameters of severe COVID-19. 

Last question: SBTC beyond ED? The authors suggest SBTC is useful to determine what could be the best setting in which to treat a patient. However, high-flow oxygen nasal cannulas (HFNCs)—and even CPAP/NIV, on some occasions—are widely used in a general ward, and do not strictly require the patient to be admitted to ICU or a high-dependency unit [7]. Unfortunately, the authors did not report if the patients were subsequently admitted in ICU or Semi-Intensive Respiratory Care Unit (SIRCU). It would have been useful to know if the SBTC was a reliable tool even in the early detection of patients requiring a high-dependency setting. 

We therefore believe that a wider study could lead to interesting results. For example, patients could be selected with stricter criteria (e.g., ruling out patients with interfering medical conditions), the test could be performed multiple times during the day to minimize the burden of anxiety, and the follow-up could be extended to more than 24 h.
